# Supplementary material for: Identification of two functional xyloglucan galactosyltransferase homologs BrMUR3 and BoMUR3 in brassicaceous vegetables
Source: PeerJ. 2020 May 14;8:e9095. doi: 10.7717/peerj.9095 (PMC7231499; doi:10.7717/peerj.9095)
Supplement: Supplemental Information 2 [file peerj-08-9095-s002.docx]

Supplementary File 2: Amino sequences of *MUR3* homologous gene

# >Arabidopsis thaliana//AtMUR3

MFPRVSMRRRSAEVSPTEPMEKGNGKNQTNRICLLVALSLFFWALLLYFHFVVLGTSNIDKQLQLQPSYAQSQPSSVSLRVDKFPIEPHAAPSKPPPKEPLVTIDKPILPPAPVANSSSTFKPPRIVESGKKQEFSFIRALKTVDNKSDPCGGKYIYVHNLPSKFNEDMLRDCKKLSLWTNMCKFTTNAGLGPPLENVEGVFSDEGWYATNQFAVDVIFSNRMKQYKCLTNDSSLAAAIFVPFYAGFDIARYLWGYNISRRDAASLELVDWLMKRPEWDIMRGKDHFLVAGRITWDFRRLSEEETDWGNKLLFLPAAKNMSMLVVESSPWNANDFGIPYPTYFHPAKDSEVFEWQDRMRNLERKWLFSFAGAPRPDNPKSIRGQIIDQCRNSNVGKLLECDFGESKCHAPSSIMQMFQSSLFCLQPQGDSYTRRSAFDSMLAGCIPVFFHPGSAYTQYTWHLPKNYTTYSVFIPEDDVRKRNISIEERLLQIPAKQVKIMRENVINLIPRLIYADPRSELETQKDAFDVSVQAVIDKVTRLRKNMIEGRTEYDYFVEENSWKYALLEEGQREAGGHVWDPFFSKPKPGEDGSSDGNGGTTISADAAKNSWKSEQRDKTQ

# >Orazy sativa//OsMUR3

MSAMRRRPVLPTHQDDMEKVGGKPPQSRLCFLATLCAMFWVLIFYFHFFVIANEPGSAGADTAAGAAASIARAELPLPEPERVSDPAVPLPPPALVSEPPPTTATVAKVEDEEKPTAVAHQEAAPRDYAFQRALKTAENKSDPCGGRYIYVHELPPRFNDDMLRECERLSLWTNMCKFMSNEGLGPPLGNEEGVFSNTGWYATNQFMVDVIFRNRMKQYECLTKDSSIAAAVFVPFYAGFDVARYLWGHNISTRDAASLDLIDWLRKRPEWNVMGGRDHFLVGGRIAWDFRRLTDEESDWGNKLLFMPAAKNMSMLVVESSPWNANDFAIPYPTYFHPAKDADVLLWQDRMRSLERPWLFSFAGAPRPDDPKSIRSQLIDQCRTSSVCKLLECDLGESKCHSPSAIMNMFQNSLFCLQPQGDSYTRRSAFDSMLAGCIPVFFHPGSAYVQYTWHLPKNYTRYSVFIPEDGVRKGNVSIEDRLKSIHPDMVKKMREEVISLIPRVIYADPRSKLETLKDAFDVSVEAIINKVTQLRRDIIEDHEDKDFVEENSWKYDLLEEGQRTIGPHEWDPFFSKPKDKGGDSTNPSTNAAKNSWKNEQRGQN

# >Brassica rapa//XM_009103789.2 BrMUR3

MTPRVAMRRRSAEAAPAEPTEKGNDGKNQNNRIYLLVSLSLLFWALLFYFHFAEKQIQLQPQPQPSSSISLRVDKFPLDPPKEKEPLVTTLPPIVEKQEFPFVRALKTADNKSDPCGGKYIYVHDLPAKFNEDMLRDCKKLSLWTNMCKFTTNAGLGPPLENVEGVFSDQGWYATNQFAVDVIFSNRMKQYKCLTNDSSLAAAIFVPFYAGFDVARYLWGYNISTRDAASLELVDWLMKRPEWEIMRGKDHFLVAGRITWDFRRLSEEETDWGNKLLFLPAAKNMSMLVVESSPWNANDFGIPYPTYFHPAKDSEVFEWQERMRNLDRKWLFSFAGAPRPDNPKSIRGQIIDQCRNSKVGKLLECDFGESKCHAPSSIMQMFQGSLFCLQPQGDSYTRRSAFDSMLAGCIPVFFHPGSAYTQYTWHLPKNYTTYSVFIPEDDIRKRNMSIEERLLQIPPEQVKIMRENVINLIPGLIYADPRSELETLKDAFDVSVEAIIDKVTRLRKNMIEGRTEYDNFVEENSWKYALLEEGQREAGGHVWDPFFSKPKPGEDSSGESNGGGTTITADAAKNSWKSEQRDKTQ

# >Brassica oleracea//XM_013742723.1 BoMUR3

MTPRVAMRRRSAEAAPTEPTEKGNEGKNQNNRIYLLVSLSLFFWALLFYFHFTEKQIQLQPQPSSSISLRVHKFPLDPPKEKEPLVTTLPPAPVIVEKQEFPFVRALKTADNKSDPCGGKYIYVHDLPAKFNEDMLRDCKKLSLWTNMCKFTTNAGLGPPLENVEGVFSDQGWYATNQFAVDVIFSNRMKQYKCLTNDSSLAAAIFVPFYAGFDVARYLWGYNISTRDAASLELVDWLMKRPEWEIMRGKDHFLVAGRITWDFRRLSEEETDWGNKLLFLPAAKNMSMLVVESSPWNANDFGIPYPTYFHPAKDSEVFEWQERMRNLDRKWLFSFAGAPRPDNPKSIRGQIIDQCTNSKVGKLLECDFGESKCHSPSSIMQMFQASLFCLQPQGDSYTRRSAFDSMLAGCIPVFFHPGSAYTQYTWHLPKNYTTYSVFIPEDDIRKRNMSIEERLLQIPPEQVEIMRENVINLIPGLIYADPRSELETLKDAFDVSVEAIIDKVTRLRKNMIEGRTEYDNFVEENSWKYALLEEGQREAGGHVWDPFFSKPKPGEDSSGESNGGGTTISADAAKNSWKSEQRDKTQ

# >Brassica napus//XM_013886954.1

MVPRVAMRRRAAEVPPTEPTEKGNGKSHTNRICLVVSLSLFFWALLLYFHFVVIGSSTNMENQIHLQPQPSSSSSTSLRIHKFDPRKEKDPLAKPILPPAPVATTSTFNPPETVEFPFVRALKTVDNKSDPCGGKYIYVHDLPSRFNEDMLRDCKKLSLWTNMCKFTTNAGLGPPLENVEGVFSDEGWYATNQFAVDVIFSNRMKQYKCLTNDSSLAAAIFVPFYAGFDVARYLWGYNISTRDAASLELVDWLTKRPEWEIMRGKDHFLVAGRITWDFRRLSEEETDWGNKLLFLPAAKNMSMLVVESSPWNANDFGIPYPTYFHPAKDSEVFEWQERMRNLDRKWLFSFAGAPRPDNTKSIRGQIIDQCRNSNVGKLLECDFGESKCHSPSSIMQMFQGSLFCLQPQGDSYTRRSAFDSMLAGCIPVFFHPGSAYTQYTWHLPKNYSTYSVFIPEDDIRKRNVSIEERLLRIPPEQVRSMRENVISLIPGLIYADPRSELETLKDAFDVSVQAVIDKVTRLRKNMIEGRTEYDNFVEENSWKYALLEDGQREAGGHVWDPFFSKPKPGEDSGNDGNGGTTISADAAKNSWKSEQRDKTQ

# >Raphanus sativus//XM_018584632.1

TPRVAMRRRSAEEGVAPTEPTEKGNEGKNQNNRIYLLVSISLFFWALLLCFHFSVLGSTNPNIPKQQIQLQPQPSQSISLRVDKFPLHPPKDRLPPAPLANSTETVKSQEFSFVRALKTSENKSDPCGGKYIYVHDLPAKFNEDMLRDCKKLSLWTNMCKFTTNAGLGPPLENVEGVFSDQGWYATNQFAVDVIFSNRMKQYKCLTNDSSLAAAIFVPFYAGFDIARYLWGYNISTRDAASLELVDWLMKRPEWEIMRGKDHFLVAGRITWDFRRLSEEENDWGNKLLFLPAAKNMSMLVVESSPWNANDFGIPYPTYFHPAKDSEVFEWQERMRNLDRKWLFSFAGAPRPDNPKSIRGQIIDQCRNSKVGKLLECDFGESKCHAPSSIMHMFQGSLFCLQPQGDSYTRRSAFDSMLAGCIPVFFHPGSAYTQYTWHLPKNYTTYSVFIPEDDIRKRNMSIEERLLQIPPEQVKIMRENVIKLIPGLIYADPRSELETLKDAFDVSVEAIIDKVTRLRKNMIEGRTEYDNFVEENSWKYALLEEGQREAGGHVWDPFFSKPKPGEDSSSEGNGGTTISADAAKNSWKSEQRDKTQ

# >Glycine max//XM_006593993.2

MRKRPDQMEKGAAAKNQNSRLCCLASLSAFFWFLLLYFHFVVLSGDDTNANSSRKNSYNNHVDLDHSTLSTTPVSVGYEPPPIHQVQASPRKIGLPDPDVRRSDADTDTPRAEKIFPFMRAMRASENKSDPCGGRYIYVHDLPSRFNEDMLKECKSLSLWTNMCKFTTNAGLGPPLENAEGVFSNTGWYATNQFAVDVIFGNRMKQYECLTNDSSIAAAVFVPFYAGFDIARYLWGYNISMRDAASLDLVHWLMKRPEWSTMNGRDHFLVAGRITWDFRRLSEEESDWGNKLLFLPAAKNMSMLVVESSPWNANDFGIPYPTYFHPAKDADVFMWQDRMRQLDRKWLFSFAGAPRPGNPKSIRGQLIDQCRRSNVCKLLECDFGESKCHSPSSIMQMFQSSLFCLQPQGDSYTRRSAFDSMLAGCIPVFFHPGSAYTQYTWHLPKNYTKYSVFIPEDDIRKRNISIEERLSQIPPEQVKIMREEVISLIPRLVYADPRSKLETLKDAFDVAVQAVIDKVTNLRKDIIEGRTDDNFIEENSWKYALLPEGEHEVGPHEWDPFFSKPKDGSGDSNDSSAEVAKNSWKNERRNQS

# >Vitisvinifera//XM_003632384.3

MRRRPTTTILPEQMDKGMPKNQQTRLCFLASLSALFWVLLLYFHFVVLGNSNVDESVQLTTIPVATQSHITSVITSPPEVTNLAKSPYPELNKETSLAQSSEEKETSHAQSSKEKETPDLEMKKESHDRELDNYPFMRALRTVENKSDPCGGRYIYVHDLPPRFNEDMLKECKSLSLWTNMCTFTSNAGLGPPLENVEGVFSNTGWYATNQFAVDVIFSNRMKQYDCLTTDSSIAAAIFVPFYAGFDIARYLWGYNISVRDAASLNLVDWLMKRPEWKIMGGKDHFLVAGRITWDFRRLTDLESDWGNKLLFLPAAKNMSMLVVESSPWNANDFGIPYPTYFHPAKDTDVLIWQDRMRKLERKWLFSFAGAPRPGNTKSIRGQIIDQCRTSKVGKLLECDFGESKCHSPSSIMQMFQSSLFCLQPQGDSYTRRSAFDSMLAGCIPVFFHPGSAYTQYTWHLPKNFSSYSVFIPEDDIRKRNVSIEERLGQIPPEQVKAMREEVISLIPRLIYADPRSKLETLKDAFDVAVQAVIGKVTKLRKDIIGGQTDDNFVEENSWKYDLLEEGQREVGPHEWDPFFSKPKDQNGDSGGSSAEAAKNSWKNEQRHQS

# >Gossypium hirsutum//XM_016854228.1

MRRRPVALAPFEAMEKGSPKNQQTRLCFLASLSAFFWIFLLYFHFVVLGRSTTIEDSVPSPPFKLVSPIVNVESIPARVTKEKPPVVKPVMNTAAEKVVAYPFMRALRTVENKSDPCGGRYIYVHNLPPRFNEDMLKECKSLSLWTNMCKFTSNEGLGPPLENVEGVFENTGWYATNQFAVDVIFNNRMKQYECLTNDSSIAAAIFVPFYAGFDIARYLWGYNISRRDAASLDLVDWLMKRPEWGIMGGKDHFLVAGRITWDFRRLTEEESDWGNKLLFLPAARSMSMLVVESSPWNANDFGIPYPTYFHPAKDEEVFVWQDRMRNLERKWLFSFAGAPRPGNPKSIRGQIIDQCRQSKVCKLLECDFGESKCHSPSSIMQMFQSSLFCLQPQGDSYTRRSAFDSMLAGCIPVFFHPGSAYTQYTWHLPKNYTTYSVFIPEDDIRKRNVSIEERLSQISPEQVKIIREAVINLIPRLIYADPRSKLETLRDAFDVAVDAVINKVTKLRRNIIQGRTEYDNFVEENSWKYDLLDEGQREVGAHEWDPFFSKPKDEQRDQSAEAAKNSWKNEQRDQS

# >Hordeum vulgare//AK361809.1

MRRRSVLPSHHDDAEKGGGKPPQSRLCFLATLCVMFWVLIFYFHFSVLAPDADAQPVAVATQARIARADLPDRVSSVPALASEPPPATLPKEEKEPVALPKEEEPVPAVLHQEEPPAAVSTVAGQEEAPAAASTVVGPEEAPAAVSNVVVQEEARAAVSTVAGQEEAPPKEYPFQRALKTAENASDPCGGRYIYVHELPPRFNEDMLRECQRLSLWTNMCKFMINDGLGPPLSNEDGVFSNDGWYATNQFAVDVIFGNRMKQYECLTKDSSIAAAVFVPFYAGFDVARYLWGYNITMRDAAPHDLVDWLRKRPEWNVMGGRDHFLVGGRIAWDFRRLTDEESDWGNNLLFMPAAKNMSMLVVESSPWNGNDFAVPYPTYFHPAKDEDVFLWQDRMRSLERPWLFSFAGAPRPGDPMSIRGQLIDQCRTSNYCKLLECDLGESKCHSPSAIMKMFQSSLFCLQPQGDSYTRRSAFDSMLAGCIPVFFHPGSAYVQYTWHLPKNYTRYSVFIPEGGVRSGNVSVEEILRSIHPDVVKQMREEVINLIPKVIYADPRSKLETLKDAFDVSVSAIINKVTQLRRDIISDSEDKDFIEENSWKYELLGQRTIGPHEWDPFFSKPKPKDMGADSGNSSAEAAKNSWKIERGDQN*

# >Zea mays//XM_008662293.3

MRRRPVLPSHHDDTDKGGGKPAPARLCFLATLCAMFWVLIFYFHFAVLSDEPAAEARIARAHTRVPEQDGAGAGASRVDLPRAEEPEDERAAGVRREVAPASYPFERALRTAENKSDPCGGRYIYVHHLPPRFNEDMLRECEKLSVWTNMCRFITNDGLGPPLGNDEGVFSETGWYGTNQFSVDVVFGNRMKQYECLTEDSSVAAAVFVPFYAGFDVARYLWGYNITTRDAASLDLVEWLMKKPEWSVMGGRDHFLVAGRITWDFRRLTEEESDWGSKLLFLPAARNMSMLVVESSPWNSNDFGIPYPTYFHPGKDAEVFLWQDRMRSLERPWLFSFAGAPRPGDPMSIRGQLIDQCRVSSVCKLLECDLGESKCHSPSTVMKMFQSSLFCLQPQGDSYTRRSAFDSMLAGCIPVFFHPGSAYVQYTWHLPKNYTRYSLFIPEDDIRSRNASIEERLKSVHPDVVKQMREDVINLIPKVIYADPRSKLETLKDAFDVSIEAIINKVTKLRRDIIAGQEDKGFVEENSWKYSLLEDGQRTIGPHEWDPFFSKPKDKGGDSGGPSAEAAKNSWKSEQRGHN

# >Nicotiana tabacum//mRNA_42310_cds

MLPPYSADESLEADETLRKPPKANSNDFLKNALSNFQYQISTHPRFWLFTFFLFFQLVVLIFTRNSPFPFSTHSPPQSLPHFPSETALFADIQKPHHDPNAIYPFGDSECEYGRVYVYNLPSKFNKDLALTTCDDLDPWKWQCGLVTNDGYGKRSTDLAGILPGNLSAAWYRTNQFSSEVIFHYRLLNYRCRTNDPDSATAFYIPFYAGQAVGKYLWTDEIENRDLLSNKVLKWVQRQKYWKRYNGSDHFLTLGRITWDFRRLGDPEKLWGSTFLNRPQMQNVSRFTIERAPWDANDISVPYPTGFHPHSEKELREWQNFVLSYNRTSLFTFIGAARGDINSDFRSRLMSYCRNESDSCRVVDCAVTQCSNGSSEIQEALLSSDFCLQPKGDSLTRRSVFDCMVTGSVPVFFWRRTAYTQYQWFLPEEPGSYSVFIDPEAVRNGTASIKEILKSYSKDQVRKMREKVVETIPRIVYARPSGGLGTIKDAFEIAVEGVLKRVKDENEWKEYVDMGS

# >Eutrema salsugineum//XM_006408893.1

MIPRVAMRRRSAEVAPTEPTEKGNGKNQTNRICLLVALSLFFWALLLYFHFVVIGSSSIDKQIQLQPSYAQSQPSSVSLRVDKFPLEPQASPSKPPKEGLVTVDKPMLPPAPVANSTSTFEPPRIVEKQEFPFIRALKTVDNKSDPCGGKYIYVHDLPSRFNEDMLRDCKKLSLWTNMCKFTTNAGLGPPLENVEGVFSDEGWYATNQFAVDVIFSNRMKQYKCLTNDSSLAAAIFVPFYAGFDIARYLWGYNISTRDAASLELVDWLMKRPEWDIMKGKDHFLVAGRITWDFRRLSEEETDWGNKLLFLPAAKNMSMLVVESSPWNANDFGIPYPTYFHPAKDSEVFEWQERMRNLERKWLFSFAGAPRPDNPKSIRGQIINQCRSSKVGKLLECDFGESKCHAPSSIMQMFQGSLFCLQPQGDSYTRRSAFDSMLAGCIPVFFHPGSAYTQYTWHLPKNYTTYSVFIPEDDIRKRNMSIEERLLQIPPEQVKVMRENVINLIPGLIYADPRSELETLKDAFDVSVQAVIDKVTRLRKNMIVGRTEYDNFVEENSWKYALLEEGQREAGGHVWDPFFSKPKPGEDSSSDGSGGTTISADAAKNSWKSEQRDKTQ

# >Lupinus angustifolius//XM_019571157.1

MRRRPVGGVLPNSTEKNSSKNHNYRLFCLASLSAFFWFLFLYFHFFILSHNRNNNNYDLAQSTKLAPSLPVIHESTPTHVTISNDSQTHSRKIGFRKSNHDRDHKRDHDHDTRIPEKKSFPFERALRTSDNKSDPCGGRYIYVHDLPSQFNEDMLRECKSLSRWTNMCKFTSNAGLGPPLENVEGVFSDQGWYATNQFAVDVIFSNRMKQYECLTQDSSIAAAIFVPFYAGFDIARYLWGYNISMRDAASLELVNWLMKRPEWSIMNGKDHFLVGGRITWDFRRLSEKESDWGNKLLFLPAAKNMSMLVVESSPWNANDFGIPYPTYFHPAKDADVFNWQDRMRQLDRKWLFSFAGAPRPDNPKSIRGQLIDQCRSSRVGKLLECDFGESKCHSPSSIMQMFQSSLFCLQPQGDSYTRRSAFDSMLAGCIPVFFHPGSAYTQYTWHLPKTYTKYSVFIPEDDIRKRNISIEERLSQIPPEQVKIMREEVINLIPRLVYADPRSKLETLKDAFDVSVQAIIDKVTNLRKDIIKGHTNKNFIEENSWKYALLDEGKHEVGPHEWDPFFSKPKDGT

# >Camelina sativa//XM_010417567.2

MIPRVAMRRRSAEVAPTEPMEKGNGKNQTNRICVLVALSLFFWALLLYFHFVVLGGSSSIDKQIQLQPSYAQSQPSSVSLRVDKFPIEPHAAPSKPPKEPLVTIDKPVVLPPAPVANSVSTFTKPPRIVESVQKQEFSFIRALKTVDNKSDPCGGKYIYVHDLPSKFNEDMLRDCKKLSLWTNMCKFTTNAGLGPPLENVEGVFSDEGWYATNQFAVDVIFSNRMKQYKCLTNDSSLAAAIFVPFYAGFDIARYLWGYNISRRDAASLELVDWLMKRPEWEIMRGKDHFLVAGRITWDFRRLSEEETDWGNKLLFLPAAKNMSMLVVESSPWNANDFGIPYPTYFHPAKDAEVFEWQDRMRNLERKWLFSFAGAPRPDNPKSIRGQIIDQCRNSNVGKLLECDFGESKCHAPSSIMQMFQSSLFCLQPQGDSYTRRSAFDSMLAGCIPVFFHPGSAYTQYTWHLPKNYTTYSVFIPEDDVRKRNISIEERLLQIPAEQVKIMRENVINLIPRLIYADPRSELETQKDAFDVSVQAVIDKVTRLRKNMIEGRTEYDYFVEENSWKYALLEEGQREAGGHVWDPFFSKPKPGEDGSNDGNGGTAISADAAKNSWKSEQRDKTQ

# >Tarenaya hassleriana//XM_010542617.2

MRRRSGGVVPTEPMEKGNGKNHNNRICLLAALSVFFWAVLLYFHFVVLGSSSIDEQNQLQPSYAQNEPSISLRVDKFLPPPEPKTKTIHPPTESLNGTVGSNLPPVLVSDTDSGSTVKLPKIGEIDVPKEQGFPFMKALRTADNKSDPCGGKYIYVHDLPSRFNEDMLRDCKKLSLWTNMCKFMTNAGLGPPLENVEGVFSNTGWYATNQFAVDVIFSNRMKQYECLTNDSSIAAAIFVPFYAGFDIARYLWGYNISTRDAASLELVDWLMKRPEWEIMKGKDHFLVAGRITWDFRRLSDEESDWGNKLLFLPAAKNMSMLVVESSPWNANDFGIPYPTYFHPAKDSDVFDWQERMRNLERKWLFSFAGAPRPDNPKSIRGQIIDQCRNSKVCKLLECDFGESKCHSPSSIMQMFQSSLFCLQPQGDSYTRRSAFDSMLAGCIPVFFHPGSAYTQYTWHLPKNYTTYSVFIPEDDIRKRNMTIEERLLQIPPEQVKIMRETVISLIPRLIYADPRSKLETLKDAFNVAVQAVIDKVTRLRKNMIEGRTEYDNFIEENSWKYELLEEGQREVGGHEWDPFFSKAKDEKDSGGGRTASSAEAAKNSWKSEQRDKS

# >Phoenix dactylifera//XM_008810225.2

MRRRPPSSSHHEEMKTNGKHPPSRLCFLATLSAVFWVLIFYFHFAVLSNNPINPASSNAQHFSKDNLPSEPLVMPHSELPETDSAPKNFPFTRALRTLENKSDPCGGRYIYVHDLPSRFNDDMLKDCRKLSLWTNMCKFMTNAGMGPPLENVNGVFSNTGWYATNQFAVDVIFSNRMKQYECLTKDSSIAAAVFVPFYAGFDIARYLWGYNISVRDAASLDLVDWLMKRPEWSVMGGRDHFLVAGRITWDFRRLTDSESDWGNKLLFLPAAKNMSMLVVESSPWNANDFGIPYPTYFHPAKDADVFVWQDRMRKLERKFLFSFAGAPRLDNPKSIRGQIIDQCKKSKVCKLLECDFGESKCHSPSSIMQMFQSSLFCLQPQGDSYTRRSAFDSMLAGCIPVFFHPGSAYTQYTWHLPRNYSRYSVFIPEDDIRKRNVSIEERLKQIPLDVVKDMREEVINLIPRLIYADPRSKLETLKDSFDVAVQAIIDKVTKLRRDIIEGHEDEDFIEENSWKYALLEEGQRTVGPHEWDPFFSKPKDGGGDSGSSSAEAAKKSWKNEQRSQS

# >Elaeis guineensis//XM_010934839.1

MRRRPPASSYHEEMEKTNDKHPPSRLCFLATLAAMFWVLIFYFHFAMLSDNPINLPERPARFHHISSNTPHLSKDQNPSEFTSSSGQHLSKDNHPSEPLVMPHPKLPDTHPAPKNYPFSRALRTLDNKSDPCGGRYIYVHDLPSRFNDDMLKDCRKLSLWTNMCKFMTNAGMGPPLENINGVFSNTGWYATNQFAVDVIFSNRMKQYECLTKDSSIAAAIFVPFYAGFDIARYLWGYNISVRDAASLDLVDWLMKRPEWSVMGGRDHFLVAGRITWDFRRLTDSESDWGNKLLFLPAAKNMSMLVVESSPWNANDFGIPYPTYFHPAKDADVFVWQDRMRKLERKFLFSFAGAPRPDNPKSIRGQIINQCKKSKACKLLECDFGESKCHSPSSIMQMFQSSLFCLQPQGDSYTRRSAFDSMLAGCIPVFFHPGSAYTQYTWHLPRNYSKYSVFIPEDDIRKRNVSIEETLKQIPSDVVKDMREEVINLIPRLIYADPRSKLETLEDAFDVAVQAIIDKVTKLRTDIIEDREDKDFIEENSWKYALLEKGQTVGAHEWDPFFSKPKDGDGDSGGSSAEAAKNSWKNEQRSQS

# >Musa acuminata//XM_018820666.1

MRRRLSASVHHEGMEKANGKPRPSRLCFLATLSAMFWIMIFYFHFTVLSSNSINNPEQSVSYSIPSKSQRISEAYEALELPKMKVQSQLKPSDQQAPEVFPFTRALQTIDNKSDPCGGRYIYVHDLPSWFNADMLRDCRKLSLWTNMCKFTGNAGLGPPLQNAEGVFSNTGWYATNQFAVDVIFNNRMKQYECLTKDSSIAAAIFVPFYAGFDIARYLWGYNISVRDSSSLELVDWLMKRPEWSVMGGRDHFLVAGRITWDFRRLTDSNSDWGNKLLFLPAAKNMSMLVVESSPWNANDFGIPYPTYFHPAKDAEVFIWQDRMRELKRRYLCSFAGAPRPGNPKSIRGQIIDQCQRSKVCKLLECDFGESKCHSPSSIMQMFQSSLFCLQPQGDSYTRRSAFDSMLAGCIPVFFHPGSAYIQYTWHLPRNYSTYSVFIPEDDIRKKNVSIEERLKQIPPDVVEIMRETVISLIPKLIYADPRYKLETLKDAFDVAVQAVIDKVTQLRRDIVEGHEDKDFIEENSWKYALLEDGQRMVGPHQWDPFFSKPKDGNGDSGTSSAEAAKNSWQNEQRSQV

# >Manihot esculenta//XM_021777263.1

MRRRPAASVPFEQMDKGTGKNQQNRLCLLASLSAFFWILLLYFHFAVLGGNNVDQSVKLEDQPLNTESKTSTLVTDARLTNTPSKTAPFIDASLKNIPSITSSQEEENFPFMRALRTIENKSDPCGGKYIYVHDLPPRFNEDMLKECRSLSLWTNMCKFTSNAGLGPPLENVEGVFSNTGWYATNQFAVDVIFSNRMKQYECLTNDSSIAAAIFVPFYAGFDIARYLWGYNISKRDAASLDLVDWLMKRPEWGIMEGRDHFLVAGRITWDFRRLTDEESDWGNKLLFLPAAKNMSMLVVESSPWNANDFGIPYPTYFHPAKDDDVFVWQERMRNLERKWLFSFAGAPRPDNPKSIRGQIIDQCKKSKVGKLLECDFGESKCHSPSSIMQMFQSSLFCLQPQGDSYTRRSAFDSMLAGCIPVFFHPGSAYTQYTWHLPKNYTTYSVFIPEDDIHKRNVSVEEVLSKIPPEQVKIMREKVISLIPGLIYADPRSKLETLKDSFDVAVQAVIDKVTRLRRNIIQGRTEYDNFVEENSWKYELLDAGQREVGAHEWDPFFSKPKDGNSDSGGSSAEAAKKSWKNEQRDQS
