# Supplementary figures and images for: Identification of two functional xyloglucan galactosyltransferase homologs BrMUR3 and BoMUR3 in brassicaceous vegetables
Source: PeerJ. 2020 May 14;8:e9095. doi: 10.7717/peerj.9095 (PMC7231499; doi:10.7717/peerj.9095)

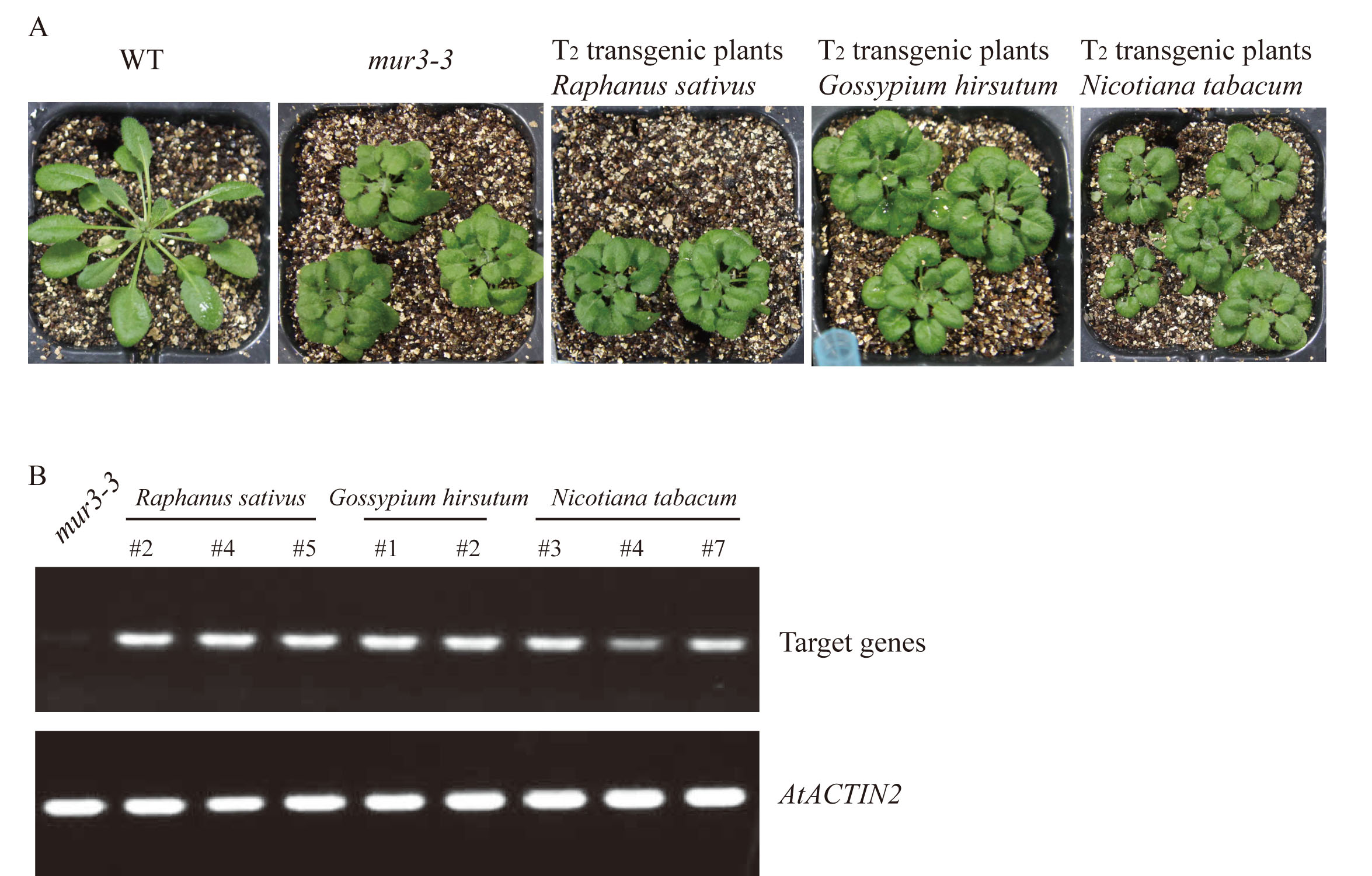

Supplement: Supplemental Information 3 — All T1 transgenic plants were verified by PCR amplification of the target genes. [file peerj-08-9095-s003.jpg]

Trans 2K  
BrMUR3com #7  
BrMUR3com #10  
BrMUR3com #11  
BoMUR3com #21  
BoMUR3com #22  
BoMUR3com #25  
WT  
mur3-3

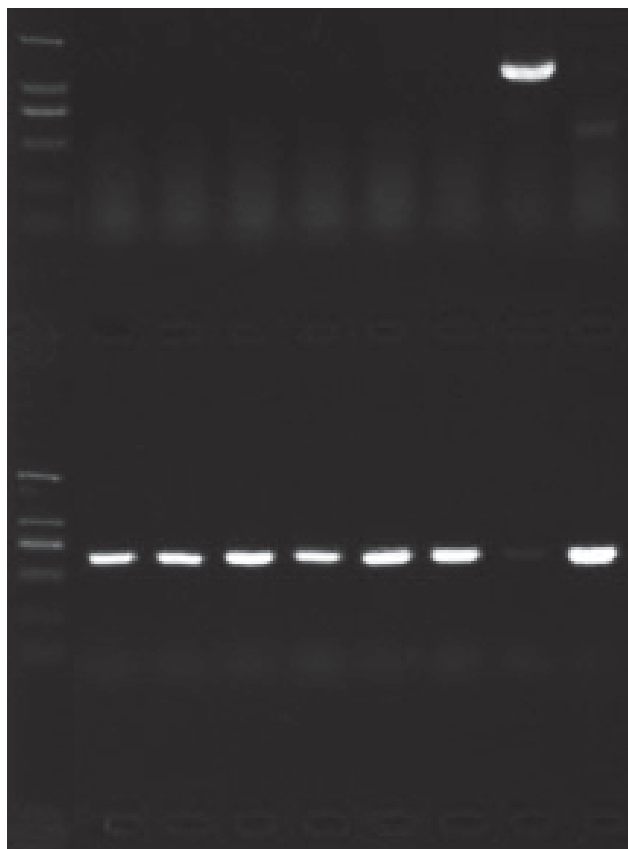

*mur3-3* LP + *mur3-3* RP

*mur3-3* RP + LBb1.3

Supplement: Supplemental Information 4 [file peerj-08-9095-s004.pdf]
